# Biomimetic synthesis technology for preparation of Fe_3_O_4_-encapsulated biochar using in highly efficient peroxodisulfate activation

**DOI:** 10.3389/fchem.2022.967589

**Published:** 2022-07-19

**Authors:** Yangyang Wang, Jianfeng Xu, Xiaoshu Wang, Tongtong Li, Gen Zhang, Zheng Yan, Jiancong Liu, Lei Wang

**Affiliations:** ^1^ School of Materials and Environmental Engineering, Institute of Urban Ecology and Environment Technology, Shenzhen Polytechnic, Shenzhen, China; ^2^ Fujian Provincial Key Laboratory of Soil Environmental Health and Regulation, Fujian Agriculture and Forestry University, Fuzhou, China; ^3^ Beijing GeoEnviron Engineering & Technology, Inc., Beijing, China; ^4^ Technical Centre for Soil, Agriculture and Rural Ecology and Environment, Ministry of Ecology and Environment, Beijing, China; ^5^ School of Ecology and Environment, Anhui Normal University, Wuhu, China; ^6^ Chinese Society for Environmental Sciences, Beijing, China

**Keywords:** biomimetic synthesis, Fe3O4-encapsulated biochar, peroxydisulfate, 2,4-dichlorophenol, DFT calculation

## Abstract

The background value of iron in red soil is superior, primarily absorbed and homogeneously encapsulated in harvested biomass. However, this property on the high-value utilization of bionic iron-encapsulated biomass remains unknown. In this study, special biochar (Fe@BC) was obtained from this kind of biomass by one-step pyrolysis method, which was further used to activate peroxydisulfate (PDS) and degrade 2,4-dichlorophenol (2,4-DCP). The results showed that Fe_3_O_4_ was formed and homogeneously embedded in biochar at 500^o^C. Comparing to catalysts prepared by impregnation pyrolysis (Fe/BC), Fe@BC exhibited excellent degradation performance (90.9%, k = 0.0037 min^−1^) for 2,4-DCP. According to the free radicals quenching studies, hydroxyl radicals (·OH) and superoxide radicals (·O_2_^−^) were the dominant reactive oxygen species (ROS) in Fe@BC/PDS system. Importantly, a PDS adsorption model was established, and the electron transport and PDS activation in the core-shell structure were demonstrated by DFT calculations. Therefore, this study could supply a high-performance catalyst and significant implications for high-value biomass utilization in red soil.

## Introduction

Red soil is widely distributed worldwide, and more than 50 million tons of iron-rich biomass is harvested through agricultural and forestry production annually. The high background value of the iron element in red soil is bioavailable, which can be absorbed during plant growth and evenly distributed in biomass. (Pi et al., 2020) Large amounts of iron element (more than 8.75%, dry weight) in biomass are not utilized effectively, e.g., Iris sibirica L. (Wang et al., 2021) However, the ignoring of the iron element in biomass needs to be emphasized to promote the sustainable and high-value utilization of iron-rich biomass. Such iron element in biomass would be biomimetic encapsulated and evenly distributed in biochar (more than 20.00%, iron) through pyrolysis procedure, the spatial distribution characteristic of iron element in biochar might bring in new function for this special material.

Generally, metal-loaded biochar is a way to enhance the catalytic performance of materials. Attributed to its reversibly electronic mobility, this material has been widely developed in energy storage, selective catalysis, environmental restoration, and so on. Sol-gel, coprecipitation, and hydrothermal method are recognized as common approaches for the metal loaded carbon. Using the impregnation-pyrolysis method to load iron-manganese oxides on biochar effectively improved the photocatalytic performance, and the degradation efficiency of naphthalene was increased by 37.9%. (Li et al., 2019) However, these methods are inefficient due to the aggregating of the metal particles, the extensive use of chemicals, and the fussy optimization strategy. (Chen et al., 2020) In contrast, as an environmentally friendly metal, iron could effectively avoid potential environmental hazards. Overall, it can be inferred that the sustainable conversion from iron-rich biomass to iron-loaded biochar can be achieved by biomimetic synthesis technology with one-step pyrolysis instead of additional loading procedures.

By biomimetic synthesis technology, a new kind of iron-loaded biochar was produced, in which the iron element was bio-inspired in biochar. Subsequently, we demonstrated the essential characterization (specie, distribution). 2,4-Dichlorophenol (2,4-DCP) is a polycyclic aromatic hydrocarbon widely derived from oil refining, coking and papermaking. At present, biochar, metal oxide, carbon nanotubes, and C_3_N_4_ were commonly used to degrade 2,4-DCP by catalytic peroxodisulfate (PDS). Furthermore, 2,4-DCP removal had been extensively studied by biocathode systems. (Chen et al., 2022) In this study, the catalytic ability of Fe@BC for the decomposition of PDS was investigated with 2,4-DCP as the target pollutant. Overall, we developed a biomimetic synthesis technology to strengthen the commercialization of the preparation of iron-loaded biochar, which could also enhance the sustainability and high-value utilization of iron-rich biomass in red soil region.

## Materials and methods

### Materials

Peroxydisulfate (Na_2_S_2_O_8_, PDS), 2,4-dichlorophenol (C_6_H_4_Cl_2_O, 2,4-DCP), sodium hydroxide (NaOH), ethanol (C_2_H_6_O, EtOH), sulfuric acid (H_2_SO_4_), tert-butyl alcohol (C_4_H_10_O, TBA), methanol (CH_4_O, MeOH), and p-benzoquinone (BQ, C_6_H_4_O_2_) were purchased from Aladdin Industrial Corporation (Shanghai, China). Hoagland’s Nutrient Solution was obtained from Xi’an Qiyue Biotechnology Co., Ltd. All chemicals were of analytical grade and did not require any further purification.

### Preparation of catalysts

Iris sibirica L. was a common emerging aquatic plant with a super-accumulation capacity for metal ions. (Wang et al., 2018) In particular, it was widely used in phytoremediation of heavy metal pollution. (Ma et al., 2017) Plants that were 6 months old and of similar biomass were selected and transplanted into the greenhouse for hydroponics. Furthermore, the concentration of iron ions were additionally added (500 mg L^−1^) during the cultivation process. The nutrient solution were completely replaced every week. The cultivation time in this experiment was 60 days.

The dried samples were pyrolyzed in a tubular retort furnace at 500°C for 2 h with a heating rate of 10^o^C min^−1^, and a N_2_ atmosphere was maintained. By calculation, the yield of biochar was only 38.76%. The obtained biochar was washed with deionized water and dried at 70^o^C for 48 h. As a control, iron-loaded biochar (Fe/BC) was prepared by impregnation pyrolysis method. Furthermore, it was guaranteed that the iron loading of Fe/BC was consistent with Fe@BC.

### Characterization and analytical method

The phase structure was determined by a Shimadzu-7000S advanced X-ray diffractometer (XRD). The microscopic morphologies of the obtained samples were recorded using a Hitachi-SU8000 scanning electron microscope (SEM). The chemical composition of obtained samples was analyzed by a an Axis-Ultra X-ray photoelectron spectroscopy (XPS). The iron concentration in Fe@BC sample was determined by an inductively coupled plasma-optical emission spectrometry.

### Batch experiments

All experiments were performed in 250 ml conical flasks with magnetic stirring (speed = 500 rpm) at room temperature (25^o^C). Fe@BC (20 mg) was added to 100 ml of solution containing 2,4-DCP (10 mg L^−1^) for saturated adsorption, and the adsorption was saturated in 90 min, and then PDS (100 mg L^−1^) was added to start the degradation reaction. At different elapsed times (up to 640 min), aliquots of 1.0 ml were extracted at given time intervals and immediately filtered through a PES filter (0.22 µM) to remove catalyst. The 2,4-DCP concentration in the obtained filtrate was further detected by high performance liquid chromatography (HPLC). In particular, quenching experiments were performed with MeOH (100 mm), TBA (100 mm), and BQ (10 mm), respectively, to evaluate the contribution of SO_4_^−^, ·OH, and O_2_^−^ in the degradation process of 2,4-DCP.

Furthermore, the degradation curve of 2,4-DCP could be linearly fitted in a pseudo-first-order kinetic mode, expressed as follows:

$$-Ln\left(C/C_{0}\right)=kt$$ (1)

where $C\left(mg\;L^{-1}\right)$ is the concentration of 2,4-DCP at t, $C_{0}\left(mg\;L^{-1}\right)$ is the 2,4-DCP initial concentration, $k\left(\min^{-1}\right)$ is the pseudo-first-order rate constant, and t (min) is the reaction time.

## Results and discussion

### Catalyst characterization

**FIGURE 1:**
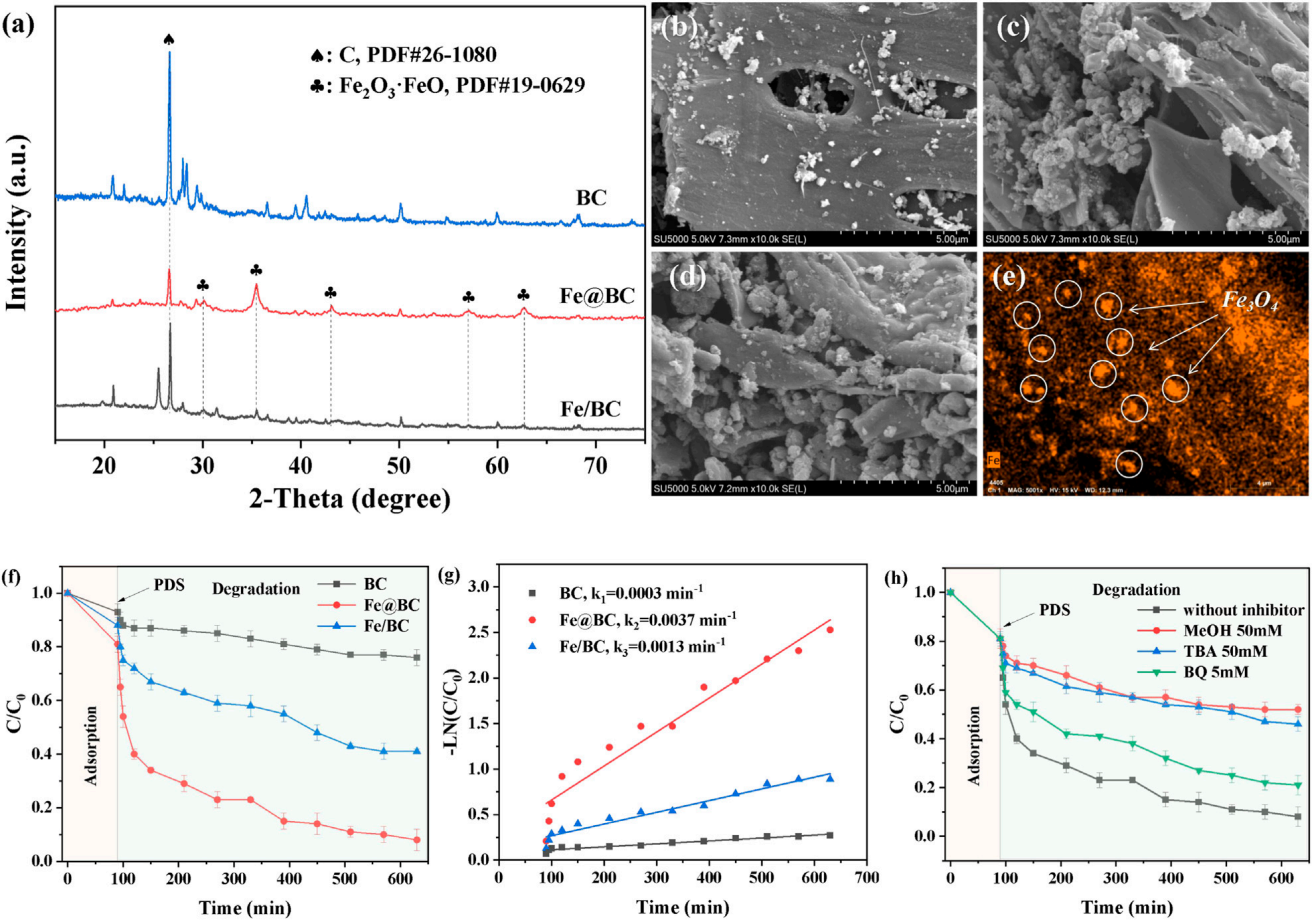
**(A)** XRD patterns of BC, Fe/BC, and Fe@BC. SEM images of **(B)** BC, **(C)** Fe/BC, **(D)** Fe@BC, and **(E)** elemental mappings of Fe@BC. **(F)** Removal efficiency and **(G)** kinetic curves of 2,4-DCP in BC/PDS, Fe/BC/PDS, and Fe@BC/PDS systems. **(H)** Influence of radical scavengers on 2,4-DCP removal in Fe@BC/PDS system.

The crystal structures of BC, Fe/BC, and Fe@BC were characterized by XRD, as shown in Figure 1A. The XRD peak at 26.6^o^ in BC was regarded as graphitic carbon structure (PDF#26-1080). In particular, the peak intensity of carbon weakened with the introduction of iron, suggesting that the introduction of iron might influence the formation of graphitic carbon. As for Fe@BC, the diffraction peaks at 18.3°, 30.1°, 35.5°, 36.9°, 43.1°, 56.9°, and 62.6° were ascribed to Fe_3_O_4_ (PDF#19-0629). (Suppiah and Abd Hamid, 2016) The strong and sharp diffraction peaks of Fe_3_O_4_ in Fe@BC indicated its high crystallinity. Usually, the presence of metals in carbon-based precursors induced the formation of carbon shells during pyrolysis process. The diffraction peaks of Fe_3_O_4_ in Fe@BC showed a obvious red shift, suggesting the formation of a core-shell structure. Furthermore, weak diffraction peaks were also found in XRD pattern of Fe/BC, which indicated that Fe_3_O_4_ could not be well crystallized by the iron-supported biochar prepared by the impregnation pyrolysis method.

The morphologies of BC, Fe/BC, and Fe@BC were observed by SEM. As shown from Figures 1B–D, the BC exhibited a porous and smooth structure surface, while numerous spherical nanoparticles were formed on Fe/BC and Fe@BC surfaces. By contrast, the distribution of spherical nanoparticles in Fe@BC was more uniform than that in Fe/BC. Furthermore, the elemental mapping image also confirmed that Fe elements were uniformly dispersed in Fe@BC. These results demonstrated that the Fe_3_O_4_ nanoparticles could be uniformly dispersed into biochar using biomimetic preparation method.

### Catalytic evaluation

The removal efficiencies of 2,4-DCP by BC, Fe/BC, and Fe@BC were shown in Figure 1E. The adsorption equilibrium between catalysts and 2,4-DCP was obtained within 90 min, and the 2,4-DCP removal efficiencies were 6.5, 11.3, and 18.8% via BC, Fe/BC, and Fe@BC, respectively. The optimal adsorption performance of Fe@BC indicated that the synergistic promoting effect between metal oxides and biochar could be enhanced by the biomimetic preparation method. After addition of PDS, the removal efficiencies of 2,4-DCP were obviously improved. The removal efficiencies of 2,4-DCP by BC, Fe/BC, and Fe@BC increased by 16.3, 46.51, and 72.1% after 640 min, respectively. A slight increase in 2,4-DCP removal was observed in BC/PDS system, suggesting that PDS could hardly be activated by BC along. Furthermore, the low removal efficiency of 2,4-DCP by Fe/BC might be due to the aggregation of Fe_3_O_4_, which weakened the accessibility of reactants towards the active sites. By contrast, Fe@BC was the most efficient catalyst, and its kobs (0.0037 min^−1^) was almost three times that (0.0013 min^−1^) of Fe/BC (Figure 1G). As mentioned above, the synergistic effects of biochar and Fe_3_O_4_ significantly enhanced the catalytic activity.

### Identification of reactive radical species

The reactive radical species (ROS) in Fe@BC/PDS system were identified by quenching experiments. Usually, methanol (MeOH) was widely used as a quencher for sulfate radicals (·SO_4_^−^) and hydroxyl radical (·OH). Tert-butyl alcohol (TBA) and p-benzoquinone (BQ) were used as scavengers for OH and superoxide radicals (·O_2_^−^), respectively. (Anipsitakis and Dionysiou, 2004). There was little difference in 2,4-DCP removal after the addition of MEOH and TBA, suggesting that OH was rapidly generated by the reaction between SO_4_^−^ and water. Furthermore, an effective inhibition of 2,4-DCP removal (12.2%) indicated that O_2_^−^ was generated in Fe@BC/PDS system. The generation of O_2_^−^ might be duo to the one-electron reaction between PFRs and soluble oxygen in solution. (Fang et al., 2015) The results exhibited that OH was the main active species, while O_2_^−^ played a minor role in 2,4-DCP removal. Specially, the generation of persistent free radicals (PFRs) could be effectively promoted by biomimetic preparation method, thereby activating PDS. (Xu et al., 2022) Furthermore, chlorine radicals were generated by the reaction between OH and Cl^−^ released during 2,4-DCP degradation.

### Reaction mechanism

**FIGURE 2:**
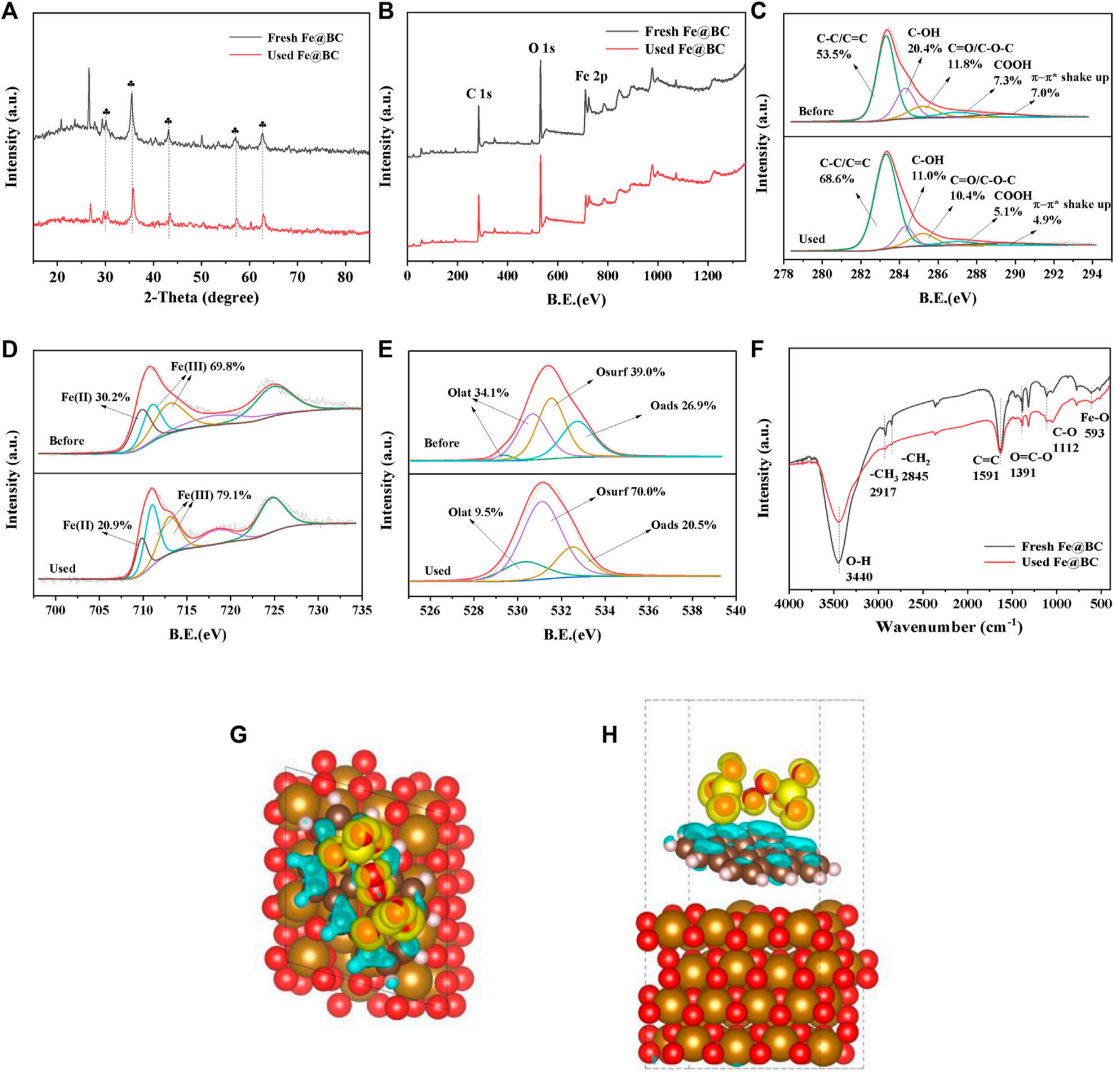
**(A)** XRD partten, **(B–E)** XPS spectra, and **(F)** FTIR spectra of fresh and used Fe@BC. **(G–H)** DFT configurations of PDS adsorption onto Fe@BC.

To explain the activation mechanism of Fe@BC with PDS, the Fe@BC samples before and after the reaction were characterized by XRD, XPS, and FTIR. As shown in Figure 2B, the peak intensity of Fe_3_O_4_ decreased slightly, suggesting the stability of Fe@BC sample. The elements of C (284.8 eV), O (531.1 eV), and Fe (711.0 eV, 724.7 eV) could be observed from the survey spectra of fresh and used Fe@BC. In Figure 2C, the species in C 1s spectra of Fe@BC were deconvoluted to C=C/C-C (284.7 eV), C-OH (285.7 eV), C=O/C-O-C (286.6 eV), COOH (288.4 eV), and π-π* shake up (290.7 eV). (Yoo et al., 2016) A obvious decrease of C-OH, COOH, and π-π* shake up was found after reaction, suggesting that these groups played an important role in PDS activation. As reported, the peak at 709.8 eV was related to Fe(II), and another two peaks at 711.0 and 713.0 eV were related to Fe(III) in Fe 2p3/2 spectra. (Lu et al., 2009) The relative content of Fe(II) decreased slightly after reaction (Figure 2D), which indicated the existence of electron cycling between Fe(II) and Fe(III). (Rong et al., 2019) The O 1s XPS spectra were fitted to four peaks with binding energies at 529.08, 530.3, 531.1, and 532.5 eV, corresponding to Olat, Osurf, and Oads (Figure 2E). (Yang et al., 2015) The relative content of Olat decreased by 24.6% after reaction, suggesting Olat participated in PDS activation. The reduction of the -OH content in FTIR spectra after reaction further proved that -OH might be an important active site in the Fe@BC/PDS system (Figure 2F). (Suding et al., 2018) Furthermore, the PDS adsorption model was constructed, and the electron transport and PDS activation in the core-shell structure were demonstrated by DFT calculations (Figures 2G,H). Also, the electron cycling process between Fe^2+^ and Fe^3+^ was further demonstrated.

## Conclusion

In this study, iron-rich biochar (Fe@BC) was prepared by biomimetic preparation method. This method not only achieved uniform distribution of Fe_3_O_4_ nanoparticles in biochar, but also enhanced the activation performance of Fe@BC for PDS. Notably, the efficient removal of 2,4-DCP could be achieved in Fe@BC/PDS system. It was found that oxygen functional groups acted as catalytic active sites. More importantly, the formed core-shell structure could effectively transfer electrons and inhibit the leaching of iron ions. This study might bring valuable insights into potential environmental applications of biomass in red soil regions.

## Data Availability

The original contributions presented in the study are included in the article/supplementary material, further inquiries can be directed to the corresponding authors.

## References

[B1] Anipsitakis G. P. Dionysiou D. D. (2004). Radical generation by the interaction of transition metals with common oxidants. Environ. Sci. Technol. 38 (13), 3705–3712. 10.1021/es035121o 15296324

[B2] Chen L. Jiang X. Xie R. Zhang Y. Jin Y. Jiang W. (2020). A novel porous biochar-supported Fe-Mn composite as a persulfate activator for the removal of acid red 88. Sep. Purif. Technol. 250, 117232. 10.1016/j.seppur.2020.117232

[B3] Chen T. Zou C. Chen F. Yuan Y. Pan J. Zhao Q. (2022). Response of 2, 4, 6-trichlorophenol-reducing biocathode to burial depth in constructed wetland sediments. J. Hazard. Mat. 426, 128066. 10.1016/j.jhazmat.2021.128066 34915250

[B4] Fang G. Liu C. Gao J. Dionysiou D. D. Zhou D. (2015). Manipulation of persistent free radicals in biochar to activate persulfate for contaminant degradation. Environ. Sci. Technol. 49 (9), 5645–5653. 10.1021/es5061512 25864382

[B5] Li L. Lai C. Huang F. Cheng M. Zeng G. Huang D. (2019). Degradation of naphthalene with magnetic bio-char activate hydrogen peroxide: Synergism of bio-char and Fe–Mn binary oxides. Water Res. 160, 238–248. 10.1016/j.watres.2019.05.081 31152949

[B6] Lu J. Jiao X. Chen D. Li W. (2009). Solvothermal synthesis and characterization of Fe3O4 and γ-Fe2O3 nanoplates. J. Phys. Chem. C 113 (10), 4012–4017. 10.1021/jp810583e

[B7] Ma N. Wang W. Gao J. Chen J. (2017). Removal of cadmium in subsurface vertical flow constructed wetlands planted with Iris sibirica in the low-temperature season. Ecol. Eng. 109, 48–56. 10.1016/j.ecoleng.2017.09.008

[B8] Pi L. Jiang R. Cai W. Wang L. Wang Y. Cai J. (2020). Bionic preparation of CeO2-encapsulated nitrogen self-doped biochars for highly efficient oxygen reduction. ACS Appl. Mat. Interfaces 12 (3), 3642–3653. 10.1021/acsami.9b19614 31894955

[B9] Rong X. Xie M. Kong L. Natarajan V. Ma L. Zhan J. (2019). The magnetic biochar derived from banana peels as a persulfate activator for organic contaminants degradation. Chem. Eng. J. 372, 294–303. 10.1016/j.cej.2019.04.135

[B10] Suding Y. Xinping Z. Yue S. Hui Z. (2018). Natural Fe-bearing manganese ore facilitating bioelectro-activation of peroxymonosulfate for bisphenol A oxidation. Chem. Eng.J.. 354, 1120–1131. 10.1016/j.cej.2018.08.066

[B11] Suppiah D. D. Abd Hamid S. B. (2016). One step facile synthesis of ferromagnetic magnetite nanoparticles. J. Magnetism Magnetic Mater. 414, 204–208. 10.1016/j.jmmm.2016.04.072

[B12] Wang Y. Lv N. Mao X. Zheng Y. Wang J. Tan W. (2018). Cadmium tolerance and accumulation characteristics of wetland emergent plants under hydroponic conditions. RSC Adv. 8, 33383–33390. 10.1039/c8ra04015j 35548110 PMC9086468

[B13] Wang Y. Wang L. Zhang Y. Mao X. Tan W. Zhang Y. (2021). Perdisulfate-assisted advanced oxidation of 2, 4-dichlorophenol by bio-inspired iron encapsulated biochar catalyst. J. Colloid Interface Sci. 592, 358–370. 10.1016/j.jcis.2021.02.056 33677196

[B14] Xu R. Li M. Zhang Q. (2022). Collaborative optimization for the performance of ZnO/biochar composites on persulfate activation through plant enrichment- pyrolysis method. Chem. Eng. J. 429, 132294. 10.1016/j.cej.2021.132294

[B15] Yang Y. Zhang S. Wang S. Zhang K. Wang H. Huang J. (2015). Ball milling synthesized MnO_x_ as highly active catalyst for gaseous POPs removal: Significance of mechanochemically induced oxygen vacancies. Environ. Sci. Technol. 49 (7), 4473–4480. 10.1021/es505232f 25760959

[B16] Yoo S. H. Jang D. Joh H. I. Lee S. (2016). Iron oxide/porous carbon as a heterogeneous Fenton catalyst for fast decomposition of hydrogen peroxide and efficient removal of methylene blue. J. Mat. Chem. A 10, 1039. 10.1039/C6TA07457J

